# Health externalities of India's expansion of coal plants: Evidence from a national panel of 40,000 households

**DOI:** 10.1016/j.jeem.2017.04.007

**Published:** 2017-11

**Authors:** Aashish Gupta, Dean Spears

**Affiliations:** a Population Studies Center, University of Pennsylvania; b Economics Department, University of Texas at Austin, 2225 Speedway, Austin, TX 78712 and Economics and Planning Unit, Indian Statistical Institute – Delhi Centre

## Abstract

Coal power generation is expanding rapidly in India and other developing countries. In addition to consequences for climate change, present-day health externalities may also substantially increase the social cost of coal. Health consequences of air pollution have proven important in studies of developed countries, but, despite clear importance, similarly well-identified estimates are less available for developing countries, and no estimates exist for the important case of coal in India. We exploit panel data on Indian households, matched to local changes in exposure to coal plants. Increased exposure to coal plants is associated with worse respiratory health. Consistent with a causal mechanism, the effect is specific: no effect is seen on diarrhea or fever, and no effect on respiratory health is seen of new non-coal plants. Our result is not due to endogenous avoidance behavior, or to differential trends in determinants of respiratory health, either before the period studied or simultaneously.

## Introduction

The effects of air pollution on health have emerged as an important issue in public economics (Currie et al., 2014). Although most of the well-identified evidence is from developed countries (Neidell, 2004, Currie et al., 2009, Lavaine and Neidell, 2013), evidence and hypotheses in the literature suggest that effects in developing countries could be even greater. This is because exposure is considerable (Arceo et al., 2016), effects could be large (Tanaka, 2015), and regulatory capacity is weak (Oliva, 2015). It is an important open question whether marginal effects at the high levels of pollution in developing countries are larger or smaller than effects at lower levels (Pope, 2015). More broadly, quantifying consequences of carbon emissions and climate change for the global poor is increasingly recognized as a major open goal for environmental and development economics (Hallegatte et al., 2016).

Against this background, health effects of air pollution *due to coal* are particularly critical for economic policy in India (Guttikunda et al., 2015), which contains 13 of the 20 cities in the world with the worst air pollution.^2^ Recent modeling exercises, using estimates from other contexts, project that health externalities of burning coal in India are very large (Cropper et al., 2012, Greenstone et al., 2015). This would be important for climate policy because India plans a large expansion of its coal power generation, which alone could make it very difficult for the world to meet emissions targets. In addition, if health externalities of coal power generation are large in India, then the full social costs of burning coal to Indians today could be considerably larger than if only future climate damages are considered. This is important because India is considered particularly vulnerable to climate change, which has suggested to some analysts that India should *accelerate* coal power generation, to develop economic security.

Despite the clear importance of the contemporary health exernalities of coal power generation in India, no well-identified estimate of this effect exists in the literature. This paper studies changes over the period of 2005 to 2012 using household-level panel survey data on 40,000 Indian households, matched to data on local changes in exposure to coal plants due to the construction of new plants. We find that increased exposure to coal plants is associated with relative worsening in respiratory health. Consistent with a causal mechanism, the effect is specific: no effect is seen on diarrhea or fever, and no effect on respiratory health is seen of new non-coal plants, such as nuclear or hydroelectric. No other trends over time suggest that local places increasingly exposed to coal plants were exposed to disadvantaged trends before or during the period studied.

This paper contributes to three active literatures. In health economics, we add to the evidence of the importance for health of air pollution, especially at the levels observed in developing countries. India is a useful setting in which to study effects of pollution because Indian migration rates are unusually low (Munshi and Rosenzweig, 2009), and migration is observed in our data, allowing us to rule out the threat of endogenously pollution-avoiding migration that has been a challenge in the literature (Moretti and Neidell, 2011). In development economics, we contribute to a growing intersection between development and environmental economics; in light of the political demand within India for increasing coal power capacity, our findings deepen the puzzle posed by Greenstone and Jack (2015) that increasingly rich people in developing countries such as India do not demand better environmental conditions. In climate economics, we provide econometrically identified estimates of effects of coal power generation in India, which imply “co-benefits” of emissions reductions and which contribute evidence of present-day social costs of India's coal expansion (Parry et al., 2015), which is also expected to have long term consequences for climate change. We use data on survey-reported costs of respiratory morbidity to Indian households to approximately quantify this aspect of the average social cost of an additional coal plant.

Section 2 presents our empirical strategy, data, and identifying assumption. Section 3 presents our main results, as well as placebo and falsification tests of our identifying assumption. In particular, Section 3.4 verifies that our results are not due to avoidance behavior or otherwise endogenous migration. Section 4 combines our effect estimates with survey data on morbidity costs to estimate an approximate lower bound of this part of the health externality costs of a coal plant. Section 5 concludes.

## Empirical strategy

### Identification strategy and regression specification

India is quickly expanding its stock of and its population's exposure to coal power plants (Dharmadhikary and Dixit, 2011), as are other developing countries (Steckel et al., 2015). India has added 82 GW of coal power capacity in the past five years, with 88 GW under construction and an additional 292 GW of coal in various stages of planning (Hannam et al., 2015).^3^ This is part of a broader effort to increase India's electrification, the effects of which have been debated in the literature (Burlig and Preonas, 2016).

Our research design is to ask whether the change in reported cough between 2005 to 2012 in India was more positive – in our case, less negative – on average in districts where new coal plants were introduced. We contrast this case with other morbidities (diarrhea and fever) which would not be expected to show an effect of coal plants and with the introduction of other types of power plant which would not be expected to have consequences for respiratory health.

**Fig. 1:**
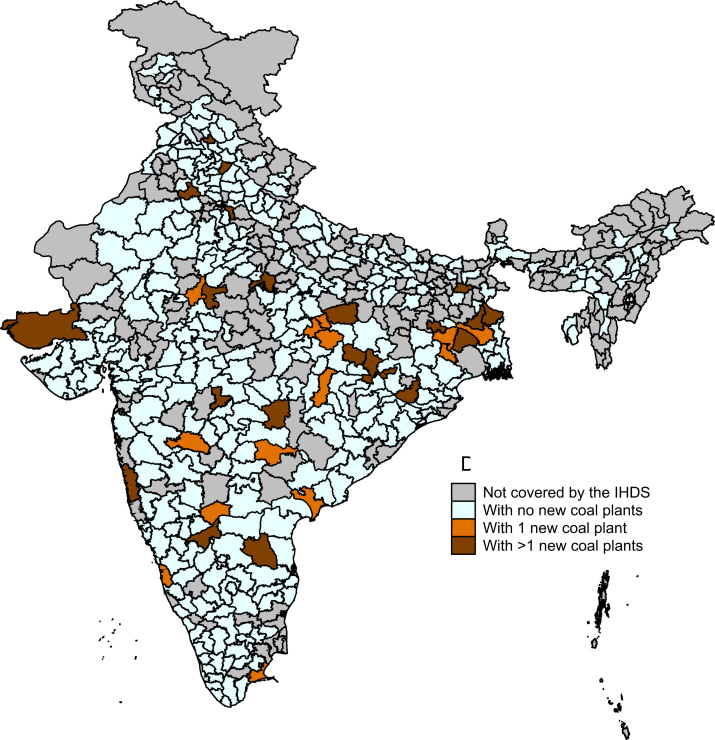
Identifying variation: Change in exposure to coal plants, 2005-2012.

The map in Fig. 1 presents our identifying variation: coal plants gained between 2005 and 2012. India is currently increasing its coal power generating capacity quickly, with an ambitious long-term plan. As the map shows, most districts did not gain a coal power plant over this time period; a small number of districts gained more than one. Increases in exposure to coal plants occurred throughout India, north and south, east and west. Districts where the survey data we use were not collected are shaded grey.

We use a difference-in-differences type identification strategy using fixed effects, a common strategy in the developed-country pollution literature (Currie and Neidell, 2005). Our use of household or other local fixed effects allows us to longitudinally study the association between changes in exposure to coal plants and changes in reported cough, while ensuring that our results are not confounded by any fixed differences across households or places, or by India-wide time trends. As we will show in detail in Section 3.4, this is an appropriate strategy in the Indian context in part because migration is very low, so our results will not reflect endogenous migration of households relative to pollution exposure.

In particular, we estimate the OLS linear probability regression:

(1) $$y_{\mathrm{ipdt}}=\beta x_{\mathrm{dt}}+\theta_{1}D_{\mathrm{ipdt}}+\theta_{2}H_{\mathrm{ipdt}}+\alpha_{\mathrm{ipd}}+\gamma_{t}+\varepsilon_{\mathrm{ipdt}},$$

where *β* is the coefficient of interest; *i* indexes individual households; *p* indexes local places or primary sampling units (PSUs) in the IHDS's two-stage sampling, either rural villages or urban contiguous blocks; *d* indexes districts, and *t* indexes time, the 2005 and 2012 rounds of the IHDS. The dependent variable *y* is an indicator for reported cough. The independent variable *x*, which varies by district-year, is the count of newly-constructed coal plants between the first and second surveys, and is therefore 0 for all districts in the initial 2005 survey round. In robustness checks, we will show results where *x* is dichotomized into an indicator for receiving a coal plant and where *x* is top-coded at four new coal plants. All specifications include the survey round fixed effect *γ* and a fixed effect *α* which will be shown as a household and as a PSU fixed effect, depending on the specification and application.

We add regression controls in three stages, to verify robustness:

• First, we show results with place and time fixed effects but no further controls.
• Next, we add *D*, a set of demographic and economic controls: consumption per capita (in log), urban residence, and household size.
• Finally, we add *H*, a set of controls for other factors that influence cough and respiratory health: cooking fuel type, use of cow dung, and whether the household has a separate kitchen or cooks outside, as well as an indicator for whether the household has electricity and the number of hours of electricity reported per day.

These controls are important because, based on the medical literature, there is no generic reason to expect households exposed to similar levels of these controls to systematically differ in their reported change in *respiratory* health in particular (without also differing in other dimensions of health).

Underlying respiratory health is in fact a continuous variable, or even a vector. We are constrained by available data to use a dichotomized indicator of survey-reported cough. Such dichotomization of the dependent variable of a regression is well-known to reduce statistical power and precision (Spears et al., 2013). Therefore, there is *a priori* reason to suspect that an otherwise similar analysis with a continuous (or more informative) measure of respiratory health could produce a more precise estimate of the effect of exposure to coal plants.

We conduct two falsification tests to verify the specificity of the relationship between reported cough and exposure to coal plants. First, in Section 3.1, we replicate our estimate of the effect on cough to produce comparable estimates of the effect on reported fever and diarrhea. Because exposure to a coal plant would not be expected to produce large effects on diarrhea and fever – especially relative to other existing sources of heterogeneity in these variables – we would not expect to find an effect. Any suggestion of an effect would be evidence of spurious differences in trends in health between districts that did and did not gain coal plants. Second, we replicate our estimate with an additional independent variable measuring the new construction of power plants other than coal. Because these other types of power plant would not be expected to cause coughs, any positive association between change in cough and gaining a non-coal power plant might suggest that our result were confounded by effects of electricity generation per se, or by the endogenous placement of power plants. Additionally, in Section 3.2 we verify the robustness of our results to different regression specifications. In particular, we use alternative versions of the independent variable; control for differential rural and urban time trends; and estimate results with a fixed-effects logit model, rather than OLS.

A limitation of our analysis is that it averages over all effects of coal plants, at the district level. Each new coal plant opening does not produce a homogeneous treatment effect: plants are of different capacity, plants could use different coal-type mixes, plants likely have different abatement technologies, plants are differentially used, and different prevailing wind patterns likely carry emissions farther (or in a more concentrated manner) in some places.^4^ Moreover, within a district, different households live closer to or further from the coal plant. Conversely, our approach implicitly assumes that coal plants have zero health effect outside of their district. Although our main approach is to average over this heterogeneity, we are able to show three plausible dimensions of differentiated effect: that the gradient is steeper in urban than rural India; that a dose-response function shows increasing effects of greater capacity; and that the marginal effect of capacity interacts with the level of capacity, such that the effect is concave (Pope, 2015). Another limitation is that we do not observe directly levels of exposure to air pollution, the mechanism that we presume links coal plants to respiratory health; instead, we study the reduced form health consequences of coal plants, which is a policy-relevant quantity for energy and environmental decision-makers.

### Data and summary statistics

Our main data source is the India Human Development Survey (IHDS), a nationally- representative panel of nearly 40,000 Indian households: the same set of households was observed in 2005 and again in 2012. The IHDS includes economic, social, and health modules. The IHDS has been widely used, such as in studies of human capital (Azam et al., 2013), education and immunization (Vikram et al., 2012), and the nutritional effects of sanitation (Duh and Spears, 2016). Our dependent variable of interest is whether the household reports any member having sought medical attention for a cough or cold within the past year.^5^ Similar data is also reported for diarrhea and for fever, which we will use in falsification tests, as we would not expect these variables to be importantly influenced by coal plants, relative to other sources of variation in them.

The IHDS is also a rich source of control variables. In particular, unlike other surveys with health information, the IHDS includes an economic consumption module, which allows us to control for changes in economic well-being and to verify that spurious economic trends are not responsible for our results. We are also able to control for whether the household has electricity and for the household size. Finally, the IHDS includes a relatively large set of variables on other sources of respiratory disease — in particular household fuel type, use of a traditional stove, and whether food is cooked in a separate kitchen or outside — so that we can account for the effect of other known influences on reported cough.

Our data on our independent variable, the construction of coal and other power plants, is compiled at the district level. These data are taken from a dataset of coal plants that India's national electricity generation monitoring agency, the Central Electricity Authority, compiled to estimate carbon emissions from India's electricity sector (Central Electricity Authority, 2015a, Central Electricity Authority, 2015b). The database contains all electricity generating plants in India, along with their date of commissioning, location, capacity, ownership, and fuels consumed.

Table 1 presents summary statistics from the IHDS and from the expansion of power plants in India. The *t*-statistic in each row tests that the average was the same in the 2005 and 2012 survey rounds. There is no standard error for additional coal plants in 2005 because all districts are zero by construction. 10.6 percent of districts gained at least one coal plant over the time period studied, and the average household in India lived in a district that gained 0.196 coal plants.

**Table 1:** Summary statistics and baseline properties by treatment status.

**Panel A: Means by survey round**					
	2005 mean	s.e.	2012 mean	s.e.	*t*-test
**independent variables**					
dichotomized gained coal plant			0.106	0.007	15.85
coal plants gained			0.196	0.014	13.94
non-coal plants gained			0.218	0.023	9.38
**dependent variables**					
reported cough	0.098	0.003	0.083	0.003	−3.90
reported fever	0.488	0.006	0.615	0.005	18.74
reported diarrhea	0.456	0.002	0.283	0.001	−8.99
**control variables**					
ln(consumption per capita)	9.589	0.010	9.909	0.009	47.63
persons per household	5.848	0.035	4.868	0.020	−34.34
urban	0.295	0.009	0.318	0.010	6.57
has electricity	0.764	0.007	0.870	0.005	21.95
hours of electricity per day	15.196	0.151	15.049	0.139	−1.01
separate kitchen	0.598	0.006	0.578	0.007	−3.45
	n=39,984		n=39,984		

**Panel B: Baseline means by treatment status, 2005 data only**					
	coal constant	s.e.	coal changed	s.e.	*t*-test

**dependent variables**					
reported cough	0.099	0.003	0.094	0.007	−0.72
reported fever	0.489	0.006	0.480	0.015	−0.56
reported diarrhea	0.045	0.002	0.048	0.005	0.50
**control variables**					
ln(consumption per capita)	9.588	0.010	9.602	0.030	0.47
persons per household	5.892	0.037	5.477	0.102	−3.84
urban	0.289	0.010	0.346	0.029	1.84
has electricity	0.760	0.008	0.795	0.018	1.77
hours of electricity per day	14.993	0.162	16.827	0.379	4.46
separate kitchen	0.602	0.007	0.565	0.016	−2.15
	n=35,763		n=4,255		

The *t*-test in panel A tests the hypothesis that the 2012 mean is equal to the 2005 mean; the *t*-test in panel B is a test for baseline balance, that households that saw an increase in their exposure to coal plants were similar in 2005 to households that did not. “s.e.” stands for standard errors, which are computed with clustering according to the survey design.

The IHDS reports that both health and economic well-being improved between the two survey waves. Overall, the fraction of households reporting cough declined by 1.6 percentage points. This improvement, we will see, was greater (more negative) in districts that did not receive a coal plant, on average. The average household consumed 37.5 percent more in 2012 than in 2005, a real growth rate of slightly over 4 percent per year. The fraction of households with electricity increased by 10.6 percentage points.

### Pre-program parallel trends

Panel B of Table 2 shows that households exposed or not exposed to an increase in coal plants were similar at baseline in consumption and in health, as measured by cough, fever, and diarrhea. The identifying assumption of this paper is the “parallel trends” assumption: it assumes that, after accounting for any observable changes, households that did not gain a coal plant form an informative counterfactual for how reported cough would have evolved over time if coal plants did not open where they did. This identifying assumption is never directly observable because it is not possible to observe what would have happened in the absence of the coal plants. However, it is possible to test that places that did and did not receive coal plants were changing according to parallel trends *before* the expansion of coal plants that we study; such a verification is standard in the literature.

**Table 2:** Pre-program parallel trends: 2005-12 increase in coal plants does not predict 1991-2001 changes.

dependent variable:	(1)	(2)	(3)	(4)	(5)	(6)
	*Δ* principal component	*Δ* IMR	*Δ* female literacy	*Δ* total literacy	*Δ* sanitation	*Δ* electricity
increase in coal plants	−0.001	4.129	0.284	0.589	−1.364	1.246
	(0.294)	(6.424)	(0.861)	(0.747)	(1.330)	(2.157)
constant	0.000	−20.111	16.404	14.029	13.434	14.649
	(0.162)	(2.753)	(0.440)	(0.367)	(0.663)	(1.515)

${}^{†}$ two-sided p<0.10, * p<0.05, ** p<0.01, *** p<0.001. Dependent variables are from the 1991 and 2001 Census of India. The “increase in coal plants” independent variable is the exact same variable as in our main results. In this case, the constant is the mean for each dependent variable among districts that did not increase in coal plants. IMR is in units of deaths per 1,000; literacy, sanitation, and electricity are in percentage points; column 1 uses the dependent variable the first principal component of the other five columns.

Table 2 presents tests that would allow the data to falsify this assumption of pre-program parallel trends, using census data from the 1990 s. In particular, the table studies changes in district-level census data between the 1991 and 2001 census of India. The table asks for each of six variables whether districts that saw an increase in coal plants *over the period 2005-2012* experienced different trends *over the earlier period 1991-2001*. The five available census variables are the infant mortality rate, female literacy, total literacy, sanitation (the fraction of households owning a toilet or latrine), and household-level electrification; from these, a sixth variable is computed, which is the first principal component of the change in these six.

The table shows that there is no evidence against parallel trends. None of these rates of change over time is different between districts that subsequently did and did not receive coal plants. Statistically insignificant differences are sometimes in the direction of greater human development and sometimes lesser, but in all cases are economically small.

## Results

**Fig. 2:**
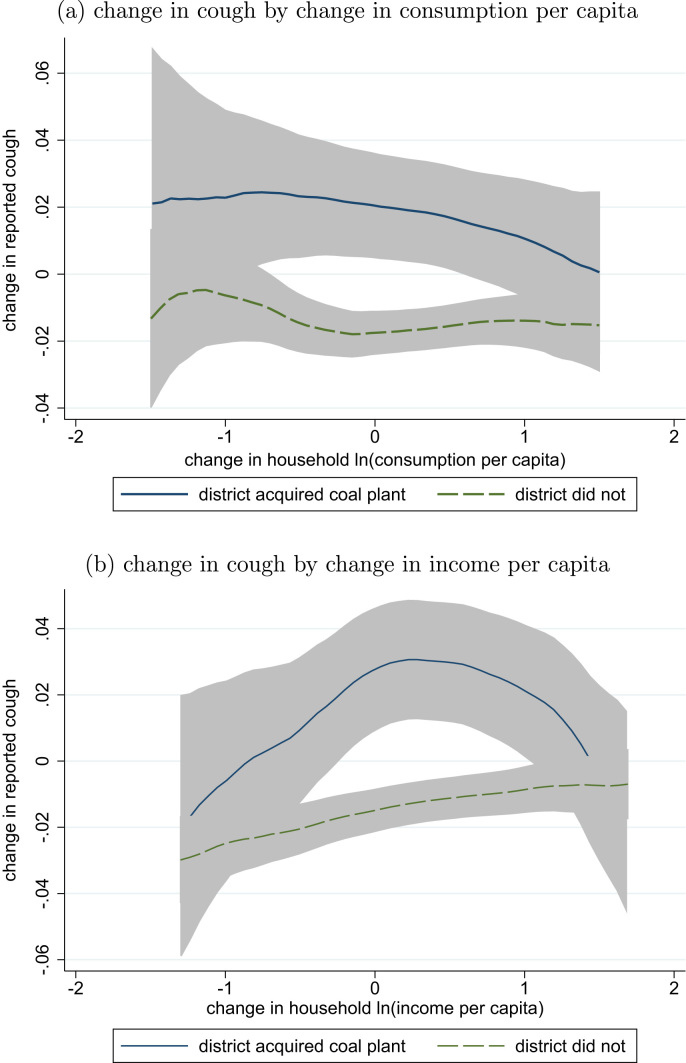
Change in reported cough for districts with and without coal expansions, by change in household economic well-being. Locally weighted regressions. Data: India Human Development Survey I (2005) and II (2012). n=79,968 observations of change over these seven years for 39,984 households.

Was the change in respiratory health different over time in places that did and did not experience increased exposure to coal plants? Fig. 2 suggests that the answer is yes. The figure splits the sample into households that live in a district which gained a coal plant between 2005 and 2012, and households that did not. The horizontal axis of Fig. 2 is the change in the log of the household's consumption per capita in panel (a) and income per capita in panel (b): households further to the right became richer by more over this time interval. The vertical axis is change in reported cough: either 1, 0, or -1, computed by subtracting the 2005 indicator for reported cough from the 2012 indicator for reported cough.

At all levels of consumption or income growth or decline, households living in places that gained a coal plant experienced a greater (more positive) change in cough than households living in places that did not gain a coal plant. The hypothesis that this paper tests is for an overall effect of exposure to coal plants on reported cough, averaging over all India. Therefore, testing for a statistically significant difference in change in cough at all levels of change in consumption or income would be a *more stringent test* than this paper's analysis is designed to answer. However, we note that the confidence intervals nevertheless show a statistically significant difference in change in cough for each level of an important interval of changes in economic well-being – in particular, this is seen at the central levels of change in economic circumstances where the distribution has the most support.

This figure, therefore, suggests evidence of an effect of change in exposure to coal plants on change in cough that does not merely reflect the confounding influence of change of economic well-being, nor of any fixed heterogeneity among households or places. The next sections verify the robustness, statistical significance, and specificity of this result.

### Main results

**Fig. 3:**
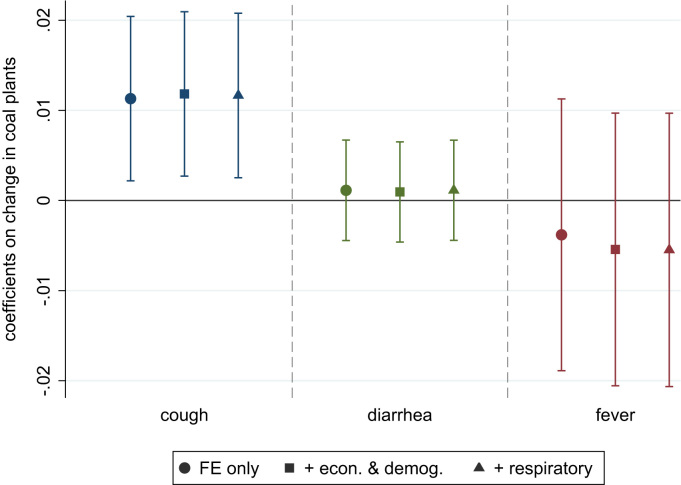
Main result: Difference-in-difference estimates of effect of coal plants on cough and on falsification outcomes. Figure plots coefficients on the count of coal plants. Each of the nine coefficients is from a separate regression where indicators for the reported symptoms listed (cough, fever, diarrhea) are the dependent variables. Each regression includes a survey round fixed effect and household fixed effects, so the graph studies change over time within households. Economic and demography controls: log of consumption per capita, urban residence, household size (count of persons) Respiratory controls: indicators for cooking fuel type, for use of cow dung, for whether the household has a separate kitchen or cooks outside, and for whether the household has electricity; the number of hours of electricity reported per day. Data: India Human Development Survey I (2005) and II (2012). Error bars are 95% confidence intervals using clustered standard errors. n=79,968 observations of change over these seven years for 39,984 households.

Fig. 3 presents estimates of Eq. (1) that constitute both our main result and a falsification test. The first three results use cough as the dependent variable. Plotted vertically are point estimates and 95% confidence intervals for β^, the additional change in reported cough associated with an additional coal plant. Estimates are approximately 0.01, suggesting that each additional coal plant added over this seven year period was associated with about one percentage point more households reporting cough. The three regression specifications and sets of controls are presented horizontally, with the circle, square, and triangle shapes. Adding controls for demographic, economic, and changes relevant to respiratory health (such as exposure to smoke and household cooking fuel) does not change the estimate of our main result. The respiratory health controls are particularly important because they reflect the set of things known in the literature to be plausibly associated with our dependent variable; any potential omitted variable would have to be uncorrelated with household fixed effects, with changes in household consumption, and with these other determinants of respiratory health. Importantly, however, these regressions with controls should be interpreted carefully, as the controls themselves could be endogenous to the opening of coal plants.

The final six results are falsification tests, using fever and diarrhea. We estimate the same regression specifications with these dependent variables substituted. For these outcomes, the point estimates are near zero, and the confidence intervals do not reject that there is no effect. Thus, as we expect, there is no evidence that coal plants are associated with these non-respiratory health outcomes.

### Robustness and falsification checks

Table 3 further verifies the robustness of our main result in alternative regression specifications. All columns include survey PSU fixed effects (for villages or small areas of urban blocks); they study change over time within places. Column 1 is a simple specification with number of new coal plants added as the independent variable of interest and with fixed effects for survey round and PSU. Column 2 interacts the survey round fixed effect with an urban indicator to allow for the controlled trend over time to differ between rural and urban places and adds a control for real household consumption per capita to account for economic changes. Column 3 adds the full set of controls from the main results figure. Column 4 replaces the independent variable with a version top-coded at 4 additional coal plants, to ensure that the results are not driven by an outlier. None of these changes to the specification importantly changes the main result.

**Table 3:** Change in reported cough and change in exposure to coal plants: Regression robustness and falsification.

	(1)	(2)	(3)	(4)	(5)	(6)	(7)	(8)
model type:	OLS	OLS	OLS	OLS	OLS	OLS	OLS	logit
additional coal plants	0.0106 ${}^{†}$	0.0110 ${}^{†}$	0.0110 ${}^{†}$			0.0116 ${}^{†}$	0.0124*	1.15*
	(0.00640)	(0.00640)	(0.00632)			(0.00633)	(0.00613)	(0.05)
additional coal plants (top-coded)				0.0119 ${}^{†}$				
				(0.00653)				
dichotomized additional coal plant					0.0267*			
					(0.0131)			
additional non-coal plants						−0.00698**		
						(0.00234)		
additional coal plants×urban							0.0461**	
							(0.0162)	
PSU (village/place) fixed effects	✓	✓	✓	✓	✓	✓	✓	✓
2012 fixed effect	−0.0178***							0.79***
	(0.00429)							(0.02)
urban×2012 fixed effects		✓	✓	✓	✓	✓	✓	
ln (consumption per capita)		−0.00351 ${}^{†}$	−0.00408 ${}^{†}$	−0.00407 ${}^{†}$	−0.00412 ${}^{†}$	−0.00416 ${}^{†}$	−0.00401 ${}^{†}$	
		(0.00211)	(0.00240)	(0.00240)	(0.00241)	(0.00241)	(0.00240)	
full set of controls			✓	✓	✓	✓	✓	
*n* (households)	79,968	79,968	79,968	79,968	79,968	79,968	79,968	79,968
primary sampling units (places)	2,435	2,435	2,435	2,435	2,435	2,435	2,435	2,435

${}^{†}$ two-sided p<0.10, * p<0.05, ** p<0.01, *** p<0.001. Dependent variable is reported cough in every column. Columns 1 through 7 are OLS linear probability models; column 8 is a fixed effect logit model reporting the odds ratio. Standard errors are clustered to reflect the survey design in columns 1 through 7, but should be interpreted with care in column 8 because classical standard errors are used. The “full set of controls” is the complete set of economic, demographic, and respiratory health controls from Fig. 3. In column 4, the independent variable is top-coded at a maximum of 4 additonal coal plants between the IHDS survey rounds. In column 7 both the coal plant independent variable and the urban indicator are demeaned to preserve comparability.

The remaining four columns make larger changes to the specification. Column 5 dichotomizes the model into a binary indicator for having gained a coal plant. The coefficient is larger in magnitude because this averages over small and large changes, and shows more precise statistical significance. Column 6 is a a falsification test: it adds a control similar to the main independent variable that is the count of *non-coal plants* built in the district over the same time period, such as nuclear or hydroelectric plants (table A4 in the online Supplementary appendix presents summary statistics describing exposure to each type of plant; of the 32 non-coal plants added during this period, 16 are hydroelectric). It does not show the same effect, or even a positive effect, indicating that our main result is not due to power plants or electrification *per se*. Column 7 interacts coal plant construction with an indicator for urban residence: the interaction is statistically significant, indicating that the association between power plant construction and cough is steeper in urban than in rural India. This is a mechanism check consistent with our main result, because the effect of pollution on population level health plausibly depends on population density, especially if coal plants tend to be located near urban areas (Peng et al., 2015). Finally, column 8 verifies that our results are robust to using a fixed effects logit model instead of an OLS linear probability model; for logit marginal effects, see figure A3 in the Supplementary appendix.

As a further robustness check, table A5 reports results at the person level, rather than the household level, using an individual-level question about “cough” from the short-term morbidity questionnaire in the IHDS individual-level recode. Results at the individual level are less precise, for reasons possibly including because they incorporate more person-level variation in health noise (such as, for example, age), but results are statistically significant and qualitatively and quantitatively comparable to our main results.

Finally, a natural question about these results is whether coal plants have cumulative effects on respiratory health, such that new plants open for a longer fraction of this period are associated with a larger difference in respiratory health. Section A4 and figure A4 of the Supplementary appendix investigate this possibility. Indeed, plants open for a larger fraction of the seven year period studied are associated with larger health consequences, which is consistent with an increasing dose-response function in cumulative exposure.

### Further verification of parallel trends

Table 2 presented evidence of pre-program parallel trends from census data, and Fig. 3 showed that change in diarrhea and fever, which would not be influenced by coal plants, did not change differentially over this period. This section further considers Table 4, which considers whether trends in other aspects of health were differential in a way that disadvantaged households which were increasingly exposed to coal plants.^6^ Table 4 estimates main regression Eq. (1) with five different binary dependent variables: an indicator for the household respondent reporting knowing a doctor or other health worker, and four indicators for the household “eligible woman” respondent correctly answering four questions about health beliefs. The four health belief questions asked about whether it is healthy to drink milk in pregnancy, whether it is healthy to feed a baby colostrum, whether smoke from a traditional stove is harmful, and whether children with diarrhea should be given more or less to drink than usual.^7^

**Table 4:** Parallel trends: Increase in coal plants predicts no disadvantage in health beliefs or service.

	(1)	(2)	(3)	(4)	(5)	(6)
	knows health provider	daily milk right	colostrum right	smoke bad right	diarrhea fluids right	LPG stove
*Δ*coal plants	000366	−0.00164	0.0281***	0.0521***	0.00835	−0.00628
	(0.00748)	(0.00758)	(0.00730)	(0.00724)	(0.00927)	(0.00778)
survey round	0.244***	0.0228***	0.0729***	0.0103*	−0.0129*	0.122***
	(0.005)	(0.00543)	(0.00475)	(0.00456)	(0.00605)	(0.007)
constant	0.073***	0.196***	0.686***	0.809***	0.649***	0.096***
	(0.007)	(0.00830)	(0.00728)	(0.00699)	(0.00936)	(0.010)

${}^{†}$ two-sided p<0.10, * p<0.05, ** p<0.01, *** p<0.001. Dependent variables, listed at top, are taken from the same IHDS panel data as our main results, and in columns 2 through 5 are indicators for correct answers to health belief questions. The “*Δ* coal plants” independent variable is the exact same variable as in our main results. LPG stands for “liquid petroleum gas,” a cleaner cooking fuel, and column 6's dependent variable is an indicator for using a clean cooking stove, including electricity. Each column uses household fixed effects, as in our main results in Fig. 2, Fig. 3.

None of these indicators show a disadvantage associated with the trend in exposure to coal plants. Columns 3 and 4 show statistically significant advantages, which, if relevant to the reported cough outcome, could bias against finding our main result. Column 4 may appear especially important^8^ because it suggests that households exposed to worsening outdoor air pollution from coal may have simultaneously been exposed to improving indoor air pollution, if this relative change in health beliefs was acted upon.^9^ However, column 6 confirms that there was no such differential trend in switching to clean cooking fuel that was associated with increasing exposure to coal plants. Instead, this change over time could plausibly be a *consequence* of the expansion of coal plants, as respondents saw household members experience worse respiratory health. Whether or not this is the case, what is clear from this table is that there is no evidence that these factors were changing in a way that relatively disadvantaged households whose exposure to coal plants increased over the period studied.

Tables A6, A7, and A8 of the Supplementary appendix expand upon this evidence for parallel trends by replicating each specification in Table 3 with each of the outcomes in Table 4 as the dependent variable. In these 48 additional regressions, the only deviation from the conclusion of Table 4 is that, in one specification, gaining a coal plant is positively associated with gaining an acquaintanceship with a doctor or health worker at the 10% level of statistical significance.

### Avoidance behavior and migration are not responsible for the result

One threat to the identification of effects of pollution that has received attention in the literature is avoidance behavior: endogenous migration to avoid pollution (Neidell, 2004, Moretti and Neidell, 2011).^10^ India is therefore an appropriate place to study effects of pollution: migration rates in India are recognized in the demographic literature to be uncommonly low (Munshi and Rosenzweig, 2009), perhaps because of the place-specific social capital of the caste system. Similarly, low mobility and residential patterns that are highly shaped by social forces (caste and religious stratification) mean that housing prices would not react to changes in pollution at the district level over a seven-year period (contrast Chay and et al., 2005).

The IHDS data that we use asks respondents how long their household has lived in the same place. Fewer than two percent of households report having moved within the past seven years, which is the interval between our two panel waves. Table 5 reports results that exclude migrant households from the sample, and that therefore can verify that no form of avoidance behavior or endogenous migration is responsible for our results. Columns 1 and 4 replicate our main result on the full sample, with and without our full set of economic, demographic, and respiratory controls. Columns 2 and 5 exclude households that report having moved within seven years before the survey wave; the result is quantitatively unchanged. Note that some of these recent movers would have moved within the same district and therefore would nevertheless be exposed to the coal plant treatment; excluding these households along with those who moved between districts makes this a more conservative test. Columns 3 and 6 additionally exclude the 71 households for which migration information is not reported. Because 98 percent of the sample reports having lived in the same place for at least the past seven years, it is unsurprising that these sample exclusions do not change our result and that we can rule out that our findings are driven by endogenous migration.^11^

**Table 5:** Results not driven by avoidance behavior or other migration.

known non-movers:	(1)	(2)	(3)	(4)	(5)	(6)
moving unknown:	✓	✓	✓	✓	✓	✓
known movers:	✓	✓		✓	✓	
	✓			✓		
additional coal plants	0.0116*	0.00985*	0.0100*	0.0117*	0.00999*	0.0101*
	(0.00466)	(0.00470)	(0.00473)	(0.00466)	(0.00470)	(0.00473)
2012 survey round	−0.0180***	−0.0177***	−0.0177***	−0.0192***	−0.0183***	−0.0184***
	(0.00311)	(0.00320)	(0.00320)	(0.00367)	(0.00378)	(0.00378)
household FEs	✓	✓	✓	✓	✓	✓
full set of controls				✓	✓	✓
*n* (included households)	79,968	78,270	78,199	79,968	78,270	78,199

${}^{†}$ two-sided p<0.10, * p<0.05, ** p<0.01, *** p<0.001. Dependent variable is reported cough in each case. “Known movers” are households that report living in the same place for only seven years or less; “moving unknown” is the small set of households for which the moving variable is missing. The dataset, the “increase in coal plants” independent variable, and set of extended controls are the exact same as in our main result Fig. 2, Fig. 3.

### Differential electrification is not responsible for the result

Another possible question about the identification of effects of pollution is whether increased exposure to new coal plants could be correlated with changes in electrification, which may in turn be correlated with health or other outcomes (Dinkelman, 2011). Note that if electrification improved health, either directly or through improving economic status, then such a threat to identification would make health costs of coal plants appear *smaller* than they in fact are. That said, recent evidence from Burlig and Preonas, 2016 suggests that electrification may not be as beneficial in rural India as is sometimes believed.^12^

Nevertheless, Table 6 tests for an association between changes in exposure to coal plants and changes in electrification, using our standard econometric strategy. We use three measures of electricity access: binary electrification, hours of electricity reported per day, and hours per day among households with electricity. None of these is associated with count or dichotomized changes in coal plants; coefficient signs are both positive and negative and magnitudes are close to zero. Therefore, there is no reason to believe that electrification is an important omitted variable.

**Table 6:** Exposure to an additional coal plant is not associated with increased electrification.

dependent variable:	has electricity {0,1}		daily hours of electricity		hours of electricity, if >0	
	(1)	(2)	(3)	(4)	(5)	(6)

additional coal plants	0.00112		0.354		0.181	
	(0.00746)		(0.219)		(0.203)	
dichotomized additional coal plant		−0.00402		0.101		−0.298
		(0.0147)		(0.435)		(0.389)
2012 survey round FE	✓	✓	✓	✓	✓	✓
household FEs	✓	✓	✓	✓	✓	✓
*n* (households)	79,583	79,583	79,505	79,505	64,901	64,901

${}^{†}$ two-sided p<0.10, * p<0.05, ** p<0.01, *** p<0.001. The dataset and the “increase in coal plants” independent variable are the exact same as in our main result Fig. 2, Fig. 3.

### The shape of the concentration-response function

A further test of the plausibility of the evidence for a causal effect is to examine the impact of the quantitative capacity of coal plants, under the hypothesis that there is a continuous dose-response function, such that people exposed to larger coal plants will be more likely to suffer from pollution. This turns out to be difficult to estimate precisely in these data due to the skew (and discreteness) of the distribution of new coal plant capacity: although the maximum district-level increase in capacity was 1,980 MW, 90% of households in the IHDS exposed to an increase were exposed to an increase of 1,000 MW or smaller, 80% were exposed to an increase of 600 MW or smaller, and over half were exposed to an increase of either 250, 300, or 500 MW.

Such a dose-response function can be estimated by inserting a continuous variable for plant capacity into our standard fixed effects regression, in place of the coal plant indicator in Eq. (1), under the assumption that pollution is positively correlated with plant capacity. Focusing on the 90% of households exposed to an increase of 1,000 or less (and all households exposed to no increase), there is clear evidence for a dose-response function, such that an increase of 1,000 MW is linearly associated with 3.4 percentage points more cough. Including the full sample, there is a statistically significant quadratic association between the increase in coal plant capacity and cough, with diminishing marginal costs of exposure to higher pollution levels. Such curvature is consistent with theories and evidence in the literature on the shape of the concentration-response function (Pope, 2015), which supports the plausibility of our results. Full details on these computations, including the histogram of exposure to increased plant capacity and regression tables, is available in Supplementary appendix section A3.

### Alternative inference

In this section, we present a robustness check of our inference, using non-parametric randomization inference (Ho and Imai, 2006, Ludbrook and Dudley, 1998). This method, which has been used in health and development economics (Bloom et al., 2006), provides a minimal-assumption answer to the inferential question of how likely it would be to see results this extreme by chance alone. Among the 389 districts in the IHDS, 46 of them gained a coal plant between 2005 and 2012. We randomly reassign 46 indicators for having gained a coal plant to the 389 districts, and re-estimate a Monte Carlo $\overset{˜}{\beta }$ using this placebo assignment of coal plants. Because this reassignment happens at the district level, it conservatively preserves any within-district correlation of outcomes. Then, we record this bootstrapped “coefficient” and replicate this randomized process 1,000 times, to produce a Monte Carlo sample of 1,000 $\overset{˜}{\beta }$ s. This provides an empirical distribution of “coefficients” against which our observed β^ can be compared. The fraction of bootstrapped “coefficients” more extreme than ours is a Monte Carlo *p*-value for the probability of seeing our result through chance alone.

**Fig. 4:**
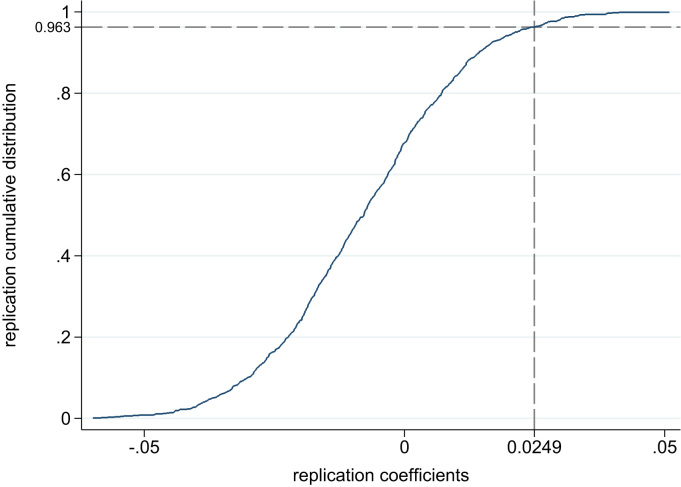
Alternative district-level randomization inference of main result: *p*=0.037. Figure plots the Monte Carlo empirical CDF of regression “coefficients” from randomization inference permutations of the assignments of the district-level coal plant increase indicator among Indian districts used in the sample. Our result is an extreme value in this set of “coefficients,” indicating that it was unlikely to arise due to chance.

Fig. 4 presents the results of this randomization inference. The figure plots an empirical cumulative distribution function of regression coefficients $\overset{˜}{\beta }$ from estimations of our main equation without controls beyond the fixed effects, and with a binary indicator for gaining a coal plan as the independent variable. Thus, the dispersion of the “coefficients” in Fig. 4 is one indication of the variation in the estimated effect size that could occur due to chance alone.

The true estimate, with a dichotomized independent variable and no controls beyond the fixed effects, is that districts which gained a coal plant over this time period experienced a more positive change in reported cough by 2.5 percentage points. Only 3.7 percent of randomization inference coefficients exceed this observed coefficient. This implies that randomization inference assigns our result a conservative district-level one-sided p-value of 0.037.

## An estimate of the social cost of a coal plant through cough morbidity

Table 7 applies the estimates of Fig. 3 and Table 3 to make an approximate calculation of the social costs of an additional coal plant, due to an increase in the short-term morbidity that IHDS respondents report as “cough or cold.” Immediately after asking about recent short-term morbidities, the IHDS asks respondents about the money they spent on medical treatment as a result of the morbidity and about missed days of work and school. Therefore, we can compute the average of these costs for the exact cases of survey-reported cough on which our estimates are based. This has the advantage of being able to study average consequences of coal plants without needing to understand precisely what sort of illness or medical symptoms respondents intend by reporting a cough. In the IHDS, the average episode of cough resulted in an out of pocket expenditure of Rs 550 on medical treatment and 4.09 days of missed work or school.

**Table 7:** Approximate implied expected discounted marginal morbidity costs of a coal plant.

Average out of pocket treatment cost per cough illness (IHDS 2012):	Rs 550
Average days missed from work or school per cough illness (IHDS 2012):	4.09 days
Average district population in persons:	2.0 m
Assumed life of coal plant:	20 years
Assumed discount rate:	3.81%
Total discounted out of pocket cost:	
Approximate market exchange rate, $1=Rs 60	$2.7 m
2011 ICP PPP for individual consumption, $1=Rs 14.0 (♣)	$11.5 m
2011 ICP PPP for health, $1=Rs 5.2	$30.9 m
Total discounted days of work missed:	
Days	1.2 m
Valued at Rs 159, at 2011 ICP PPP for individual consumption (♠)	$13.7 m
Total cost: (♣+♠=)	$25.2 m
Total cost per person-year of exposure:	$0.9

Notes: This table ignores any costs of illness beyond (♣) out of pocket costs of medical treatment for “cough” and (♠) a valuation of missed days of work and school for “cough.” Following Table 3, these computations use an effect size of β^=0.01. These computations ignore future population growth, which would increase costs by increasing the number of people exposed to pollution. Work days are conservatively valued at Rs 159 because this is the wage paid by NREGA (a national workfare program) in Madhya Pradesh, the state where this wage is lowest. Note that, at this discount rate over 20 years, the average district experiences 27.7 million discounted person-years of exposure to the coal plant.

We can produce an approximate total cost (due only to these two factors) of this health externality of an average coal power plant by linearly exploiting our estimate of the average linearized effect of a coal power plant on residents of the plant's district. This estimate, like our regression results, will ignore any consequences of the power plant for people living outside of its district, and therefore may be an underestimate. The average Indian district has just under 2 million people; if we assume a coal plant will last for 20 years and discount the future at the 3.81% rate at which the Government of India borrows money,^13^ then we find that the average coal plant will cause about 28 million discounted person-years of exposure. Combined with our cost estimates, this yields $11.5 m in out of pocket treatment costs^14^ and $13.7 m in a conservative valuation of foregone days of work at the lowest wage paid by a government workfare program for poor rural Indians, for a total of $25.2 m for each additional plant.

This figure must be regarded as highly approximate, not least because it ignores any social value of illness itself or of other consequences of exposure to coal pollution beyond survey-reported “cough.” It deliberately values foregone labor time highly conservatively. However, the value of this figure is that it suggests that it could be the case that even if the private cost of an alternative to a coal plant were several tens of millions of dollars more expensive, the total social cost of the alternative could be lower.

Both the out of pocket costs and the loss of work days have highly skewed distributions in the survey, with many respondents reporting zero or low costs and a few reporting large costs. Such skew is common in health cost data, such as in the RAND health experiment (Manning et al., 1987) or the Oregon health insurance experiment (Finkelstein et al., 2012). If we use the median out of pocket cost and loss of work days, rather than the mean, we sacrifice this information about very high costs (which may be a large fraction of the total) in order to use a more well-behaved statistic than the mean. Using these medians, we arrive at a total cost of $9.8 m, rather than $25.2 m at the means.

As a final note, we emphasize that it is not the goal of these approximate computations – which attend to only one cost of coal plants in India as they were operated over the period studied – to make an all things considered recommendation about energy or environmental policy. However, costs such as in Table 7 should be considered in comparison with costs of pollution control measures for coal plants, costs of non-coal energy sources, or the overall social cost of energy consumption. As one illustrative computation of the costs of pollution controls, note that “capital costs of wet scrubbers^15^ range from $100 to $200/KW;” ignoring continuing operating costs, this suggests a capital cost of $7 m to $13 m^16^, which is comparable in magnitude to the totals in Table 7. Even if health externalities of coal consumption are quantitatively modest, they could cause renewable energy sources to be socially preferred if the difference in private costs becomes small due to declining costs of renewables. Investigating these policy implications quantitatively is left for future research.

## Conclusion

Indian districts that gained coal plants from 2005 to 2012 also experienced a relative increase in reported cough. This result is robust to a range of respecifications (including for many of the factors known to influence respiratory health), and is not found for other non-respiratory diseases or for non-coal plants. This finding is therefore indicative of health externality effects of coal power plants in India. The fact that this effect is an externality – a consequence for people other than the decision-maker – is a classic rationale in public economics for corrective government action.

In policy discussions of the social costs and benefits of coal power generation in India and its effects on climate change, the argument is often made by Indian policy-makers that the benefits to Indians today in the form of economic development outweigh the costs to future Indians of promoting climate change. However, recent studies that attempt to quantify the full present-day social costs of coal suggest that current externalities may be strong enough to justify an optimal amount of coal consumption that is considerably below what is observed (and than the increase in coal use that is planned), even ignoring costs to future populations (Parry et al., 2015, West et al., 2013). Fully assessing this possibility is well beyond the scope of this paper's documentation of a health effect: this paper cannot assess whether, all things considered, the best policy would mandate pollution control technology or non-coal fuel sources. However, the evidence in this paper adds to this growing literature indicative of important present-day negative externalities of coal. One policy implication for policy-makers internationally is that supporting investment in non-coal and other renewable energy capacity in India could be an effective form of both longer-term climate policy and nearer-term development and health aid (Somanathan, 2015).

## Appendix A Supplementary data

Application 1

## Footnotes

2 According to World Health Organization data on PM ${}_{2.5}$ concentration.

3 These figures are from personal communication with Phillip Hannam, reflecting an updating of data and methodology from Hannam et al. (2015).

4 Other papers in the literature, studying other contexts, can overcome these limitations: for example, Clay et al., 2015 exploit geographic distance, and Schlenker and Walker (2015) exploit variation in wind.

5 Like in any study of retrospective, self-reported health, we cannot observe precisely which biological symptoms respondents intended in response to this question. The Hindi word for cough is *khansi* and for cold is *zukam*; it is very common for people to conflate these concepts and refer to having *khansi-zukam* or literally “cough-cold,” which may be what is intended by the English translation of the questionnaire (48% of interviews were conducted in Hindi, about 1% were conducted in English, and many of the other interviews were conducted in languages similar to Hindi with a comparable construction). A member of the IHDS field team with whom we discussed this question found this interpretation plausible. For this reason, throughout the rest of the paper, we refer to this variable as simply “cough.” Supplementary appendix table A5 reports results for a separate individual-level question for which the English questionnaire only mentions “cough.”

6 A related question is whether there is any evidence against non-parallel trends in alternative indicators of household socioeconomic status: table A3 in the Supplementary appendix substitutes several SES measures in as the dependent variable of Eq. (1) and finds that change in none of income per capita, having a literate adult, assets per capita, number of rooms, having electricity, or having piped water is predicted by gaining exposure to coal planes over this period.

7 The IHDS also asked further health belief questions, but these were not available in the panel; for example, a question about what influences the likelihood of pregnancy was asked about only in one round.

8 However, the coefficient becomes less than one-third as large if the 11% of respondents who answered that they do not know are excluded, suggesting that this positive coefficient is fragile and should probably be interpreted as an approximate zero. More importantly, it is of course also the case that this result could merely reflect multiple inference.

9 We can probably not be confident that these beliefs would have been acted upon: literature suggests that the adoption of clean cooking fuel in India is slowed by the fact that men typically make purchasing decisions such as for LPG, while women bear the cost of traditional fuels (Kishore and Spears, 2014); in this case, women answered this survey question. In any event, our main results below control for a complete vector of properties of households' fuel type and use.

10 Migration is a particularly extreme form of avoidance behavior. Smaller behaviors such as wearing masks to protect against pollution are only now appearing among the elite in India's metro cities in 2016, and would have been very rare in 2012. In Table 4, we have already seen that switching to clean cooking fuel, which may be one form of small avoidance behavior, was uncorrelated with the expansion in coal plants. Nor was there any difference-in-difference association between coal exposure and ownership of a pressure cooker, which requires less fuel to be burned to cook (*p*=0.81, when used as the dependent variable in main Eq. (1)).

11 If this is tested formally by introducing into column 1 an interaction between coal exposure and an indicator for “known non-mover,” there is no interaction: *p*=0.61 and the 95% confidence interval on the interaction spans from -0.36 to 0.21, well beyond the magnitude of the main coefficient.

12 As another matter, the authors’ limited qualitative fieldwork near coal plants in north India suggests that there is no generic expectation of an improvement in electrification of nearby villages. Instead, electricity is contributed to large grid networks that extend well beyond district boundaries. For more details, see section A1 of the Supplementary appendix.

13 Because many of these expected episodes of cough will occur in the future, a lower discount rate would result in a higher estimated social cost of a coal plant.

14 The precise figure is sensitive to the purchasing power parity exchange rate used; using the 2011 ICP exchange rate at the health basket of goods, instead of individual consumption overall, would increase our final estimate of the social cost by about $19 m.

15 This paper does not consider the health consequences or effectiveness of any pollution controls.

16 Supplementary appendix table A4 reports that the average new plant we study has a capacity of 65.7MW. $65,700\mathrm{KW}\times \frac{\$200}{\mathrm{KW}}=\$13.1\;m$.

Appendix A Supplementary data associated with this article can be found in the online version at doi:10.1016/j.jeem.2017.04.007.
