# Supplementary material for: Health externalities of India's expansion of coal plants: Evidence from a national panel of 40,000 households
Source: J Environ Econ Manage. 2017 Nov;86:262–76. doi: 10.1016/j.jeem.2017.04.007 (PMC5669305; doi:10.1016/j.jeem.2017.04.007)
Supplement: Application 1 [file mmc1.pdf]

A1 Qualitative evidence on electrification near coal plants

One important question about the results is whether electrification near coal plants could be a mechanism or an omitted variable. However, Table 6 of the main text showed that households living in districts that gained a coal plant were no more likely to become electrified than households in other districts. This makes sense because electricity from coal plants are contributed to grids that are much larger than individual districts.[1]

In part to further understand this question, in August of 2016 one of the authors (Spears) visited people near two coal plants in northern Indian plains, in one urban site and one rural site.[2] These areas are far from coal mines; coal is brought on trains.

People living near the coal plants emphasized considerable health and other costs of both air pollution and noise pollution. In multiple instances a respondent pointed to the interviewer's white shirt and suggested the coal smoke would make it black, especially if hung up on a line. But as much as the respondents minded the smoke, an equally common objection was that the coal plant hired either low-skilled migrant laborers or high-skilled office workers who had passed official exams — but did not give jobs to local people.

Certainly these respondents were not thinking of a difference-in-difference of their electricity supply over time relative to households elsewhere. But the clear belief of these households was that they did not receive more electricity than others. In neither case did the people living near the coal plant have 24 hours of electricity; they had daily cuts. Most respondents believed this was because power was sent through wires to distant cities, but some considered the coal plant defective ("It is like my old motorcycle, it just cannot go so fast"). One household had recently — despite the fact that the visibly nearby thermal plant was old — paid 300 rupees for an electricity connection box that powered two bulbs and a fan; it was not offering electricity at the time of the interview. Respondents emphasized that electricity was important ("electricity is life"); several suggested that they would have no objection to the coal plant if they, too, received electricity.

[1] Relatedly, Granoff, et al. (2016) have recently argued for the case of India, that "adding new coal-fired capacity has not led to many new connections for [nearby] Indians."

[2] Of course, these interviews were conducted much closer to the coal plants than the average person living in the same district, the unit of the independent variable of our main analysis.

A2 Changes in economic-well being between IHDS rounds

An important literature in development economics asks about the economic consequences of changes in electrification (Dinkelman, 2011; Burlig and Preonas, 2016). This paper has considered closely the question of whether an additional coal plant increases electrification for households *in its district* relative to the change over the same time period in other districts, and has not found evidence that it does (see table 6 of the main text and section A1 of this appendix). Nevertheless, the question is of such importance — and economic status is sufficiently critical as a potential omitted variable — that it is worth a closer look at whether households in the IHDS that gained electricity over this time period had differentially worse economic outcomes. This section reports evidence that they did not, a conclusion that would be consistent with Burlig and Preonas, worth quoting in full, who study India over a similar time period: "We find a substantial increase in electricity use, but we can reject even modest effects on labor markets, asset ownership, housing characteristics, and village-wide outcomes." If so, electrification may be unlikely to be an important omitted variable in our analysis.

Table A1 reports regressions of household income and consumption per capita on electrification, using the same difference-in-differences strategy as in our main analysis of coal plants.[3] The result depends on whether income and consumption are entered in logs or in levels, which suggests that the levels estimates may be influenced by large outliers. The credibility of our main estimates may be threatened if people who were exposed to more coal plants also became poorer over time (because poverty could contribute to poor health — although it is unclear why it would only be *respiratory* health) but there is no consistent evidence this is the case. Because it is consistent with a range of standard results and functional forms from consumer preference theory (Deaton and Muellbauer, 1980), it is customary to study total expenditure in log form, not levels. Therefore, if columns 1 and 3 are preferred, we conclude that if anything the association goes the other way.

One might still wonder whether the negative sign in the levels specifications is evidence that household that gained electrification became *poorer* from 2005 to 2012. Figure A1 documents that this is not the appropriate interpretation of these results. Households are categorized by their electrification status in 2005 and, for households without electricity in

[3]The regression equation is:

$$income_{it} = \beta electrified_{it} + \alpha_{it} + \gamma_t + \varepsilon_{it},$$

where γ is an indicator for the second survey rounds, and α are household fixed effects.

2005, whether they gained it by 2012. On average, households in all three categories became richer between 2005 and 2012, visible in the greater height of the 2012 light grey bars. However, the increase in income and consumption (levels) for households that gained electricity was smaller than the increase for households which had electricity the entire time, perhaps because of large outliers in this group. Because the large majority of households whose electrification status did not change already had electricity, these facts imply that the positive economic improvement in households that gained electrification was smaller, on average, than the improvement in households whose status did not change. This would result in the negative apparent difference-in-differences coefficient seen in columns 2 and 4. In short, therefore, the IHDS gives no reason to believe that households that gained electrification from 2005 to 2012 became poorer over time, and little evidence of much of a robustly consistent, statistically significant intertemporal association between electrification and change in economic well-being at all. Because our main results are unchanged by controlling for economic status, and are apparent at all levels of rich and poor (see figure 2 of the main text), there is no reason to believe it is an important omitted variable.

A3 The shape of the concentration-response function

If our main results indeed reflect an effect of exposure to coal plants on respiratory health, then two further results would be expected. First, there should be a positive gradient to the dose-response function, or in this case pollution concentration-response function: assuming that the pollution from a coal plant is positively associated with its electricity generation capacity, exposure to a greater increase in coal plant capacity should be associated with a greater relative increase in poor respiratory health. Second, the shape of this function – in addition to being positively-sloped – should match the shape described by evidence in the literature. Pope et al. (2015) argue that the concentration-response function for air pollution has a concave shape, such that further increases in pollution cause less marginal harm, so policy might efficiently focus on reducing lower levels of harm first.

The discrete nature of coal plants makes the shape of the concentration-response function difficult to estimate precisely. The histogram in figure A2 plots the empirical density of households in our data, based on the size of the increase in coal plant capacity to which they are exposed (89% of households, exposed to zero increase, are excluded). Two features of the data are visible: the distribution is discrete, and it is skewed. Among the households exposed to an increase 90% of households in the IHDS exposed to an increase were exposed to an

increase of 1,000 MW or smaller, 80% were exposed to an increase of 600 MW or smaller, and over half were exposed to an increase of either 250, 300, or 500 MW. This distribution cautions modestly in our ability to estimate the shape of the health response to an increase in exposure above 1,000 MW.

Table A2 presents estimates of the concentration-response function, computed by replicating our main regression equation with change in coal plant capacity substituted for the indicator for an increase in coal plants. Both predictions based on the literature are verified in our data: there is a continuous dose response function, and it curves concavely at higher levels of exposure. Thus, comparing columns 1 and 2, the linear coefficient becomes smaller when larger changes in exposure are included. The quadratic term in columns 3 and 4 use the full sample to test for this curvature explicitly; it is similar with or without the full set of regression controls from the main results. Finally, column 5 verifies that the increasing, concave shape of the ln function fits the data.

A4 Interaction with time of plant opening

We study the change over a seven year period, from 2005 to 2012. People who are exposed to new coal plants for a longer fraction of this period may experience cumulative effects. We test for such effects — in the spirit of a mechanism or plausibility check — by interacting our independent variable of interest with a scaling factor from 0 to 1 representing the fraction of the 2005 to 2012 period during which a new plant has been open in the district, at the month level. So, if the fraction is 1, the plant was opened during the month of the 2005 IHDS; if the fraction is 0 the plant was opened during the month of the 2012 IHDS. We insert this as an interaction into our main regression equation:

$$y_{ipdt} = \beta_1 x_{dt} + \beta_2 f_d \times x_{dt} + \theta_1 D_{ipdt} + \theta_2 H_{ipdt} + \alpha_{ipd} + \gamma_t + \varepsilon_{ipdt}, \tag{A1}$$

where f is the fraction defined above. It has a d subscript only and does not enter additively because it is absorbed into the district fixed effect as a property of the district ("for how long was this district exposed?").

Figure A4 plots results, by plotting the predicted effect $\hat{\beta}_1 + \hat{\beta}_2 \times f_d$, where f_d has been scaled for ease of interpretation to the years 2005 to 2012. There is a clearly visible interaction, where the effect was largest in districts exposed for most of the period, there is essentially no effect of the newest coal plants, and the effect at the average duration is similar in magnitude to those in our main results. The three sets of controls used match those in

the main results figure 3. Clustered p-values are included for the test that the interaction is statistically significant; in all three cases it is at conventional levels. Therefore, these results are consistent with exposure to coal plants having an effect that is at least in part cumulative.

References

Burlig, Fiona and Louis Preonas (2016) "Out of the Darkness and Into the Light?: Development Effects of Rural Electrification in India," working paper, University of California, Berkeley.

Deaton, Angus and John Muellbauer (1980) "An almost ideal demand system," *The American economic review*, Vol. 70, pp. 312–326.

Dinkelman, Taryn (2011) "The effects of rural electrification on employment: New evidence from South Africa," *The American Economic Review*, Vol. 101, pp. 3078–3108.

Granoff, Ilmi, et al. (2016) "Beyond coal: Scaling up clean energy to fight global poverty," position paper, Overseas Development Institute.

Pope, C Arden, III, Maureen Cropper, Jay Coggins, and Aaron Cohen (2015) "Health benefits of air pollution abatement policy: Role of the shape of the concentration–response function," *Journal of the Air & Waste Management Association*, Vol. 65, pp. 516–522.

Figure A1: Change in income and consumption, by change in electrification

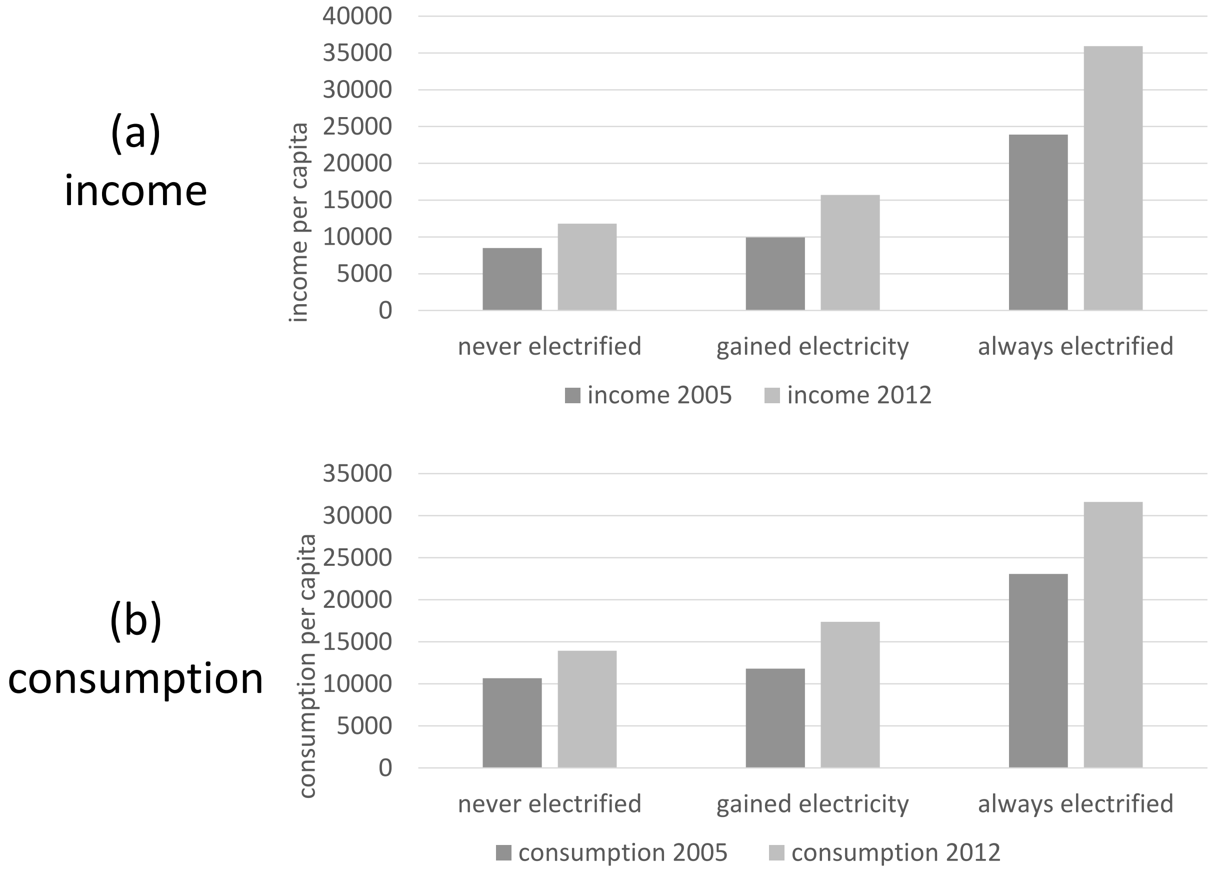

Figure A2: Distribution of exposure to increase in coal plant capacity

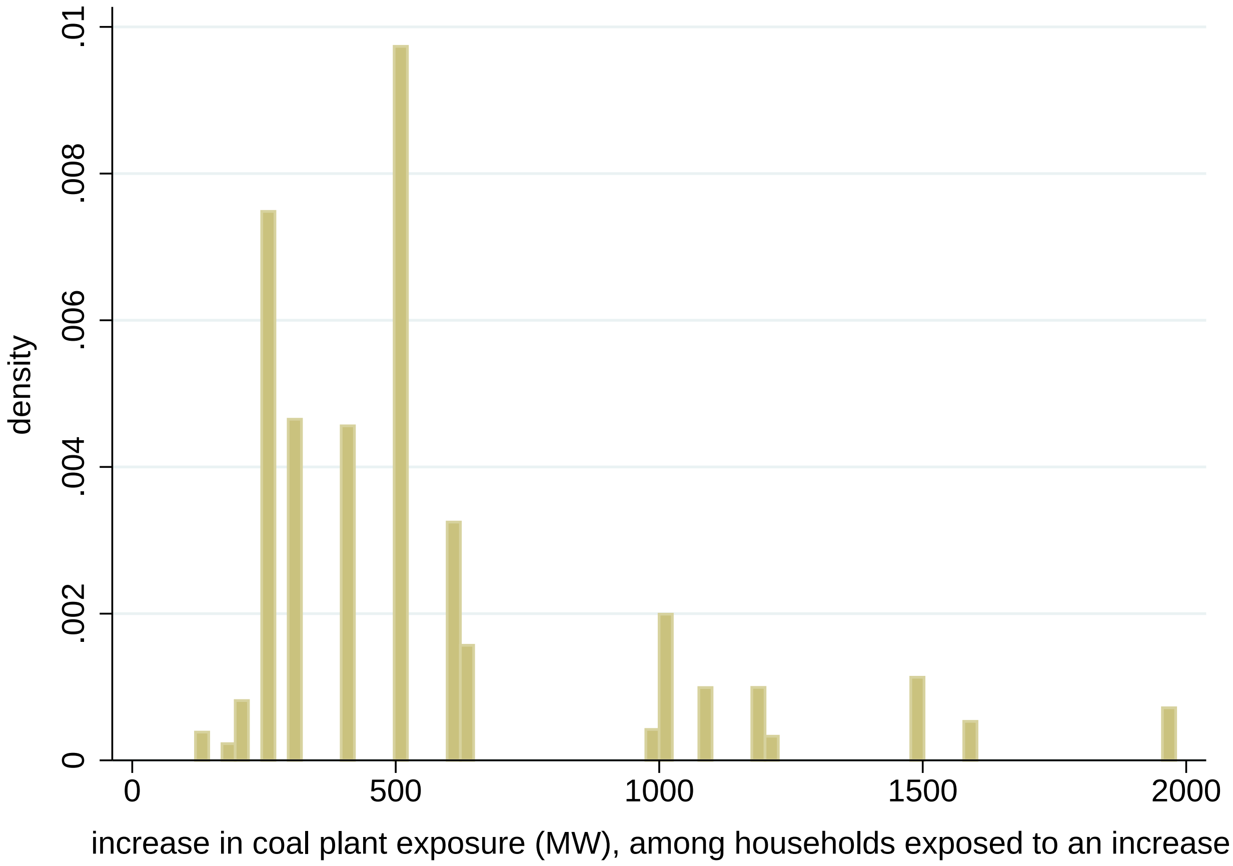

The point mass of 89% of households whose exposure to coal plants did not increase is excluded from the figure.

Figure A3: Marginal effects of exposure to coal plants, from fixed effects logit model

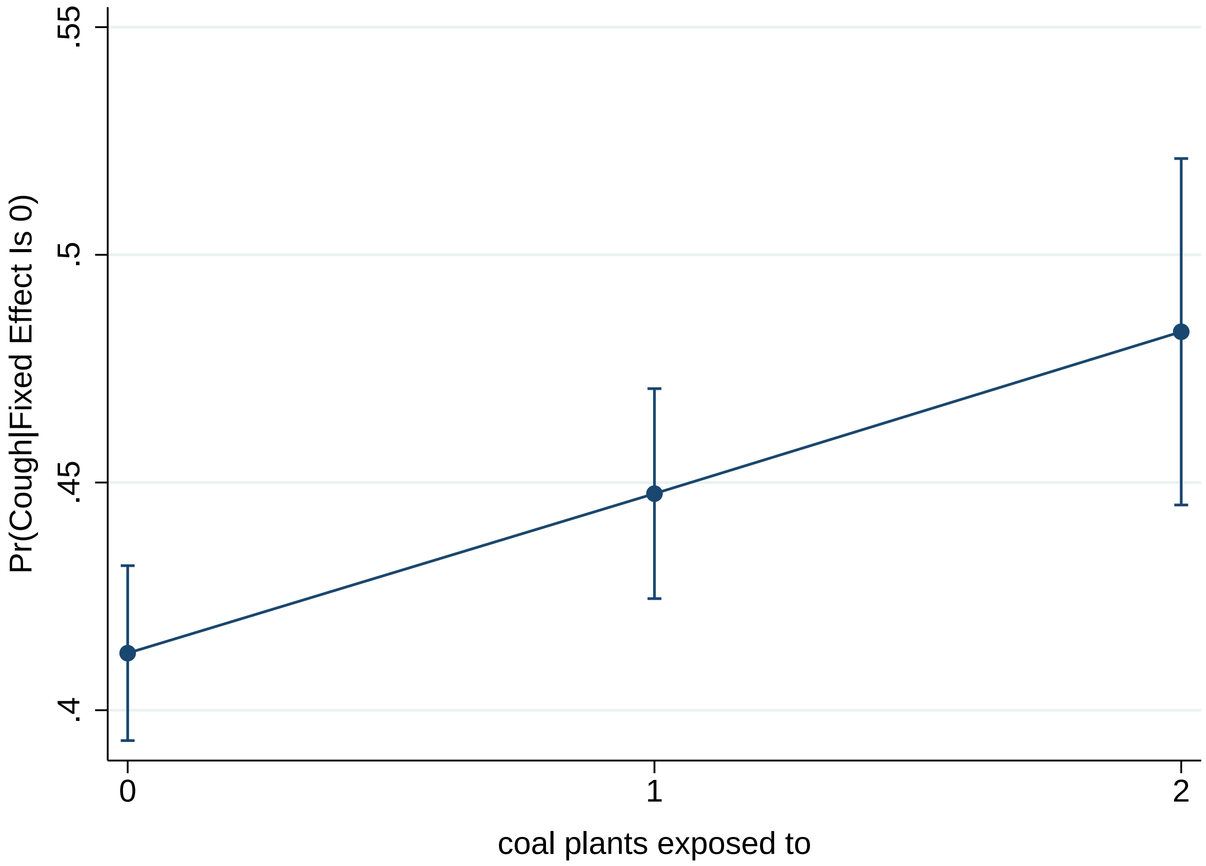

Margins plot corresponds with column 8 of table 3 from the main text. Margins are computed at the mean of the survey round fixed effect (near 1.5) and with PSU fixed effects set to zero. 95% confidence intervals are plotted using the delta method standard error.

Figure A4: Linear interaction: Predicted effects of coal plants on cough is greater the longer respondents have been exposed

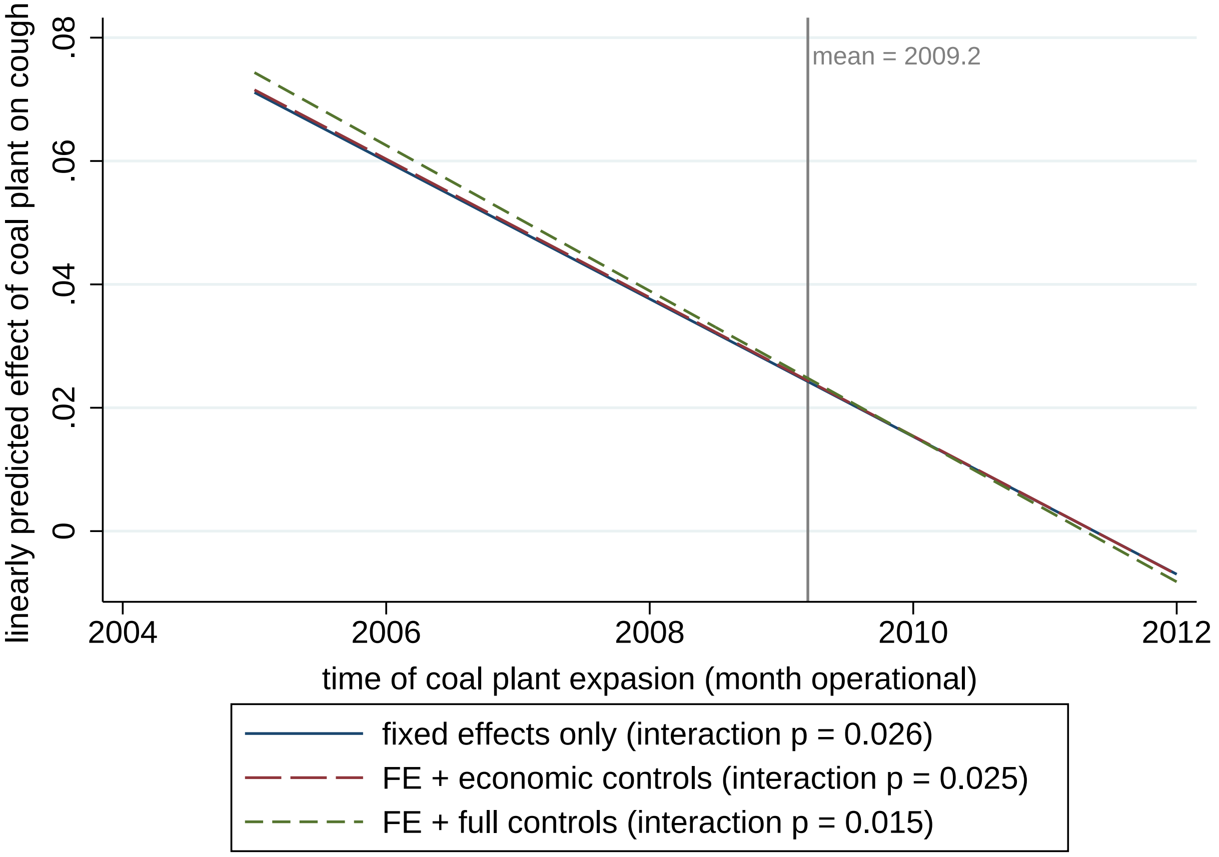

Plots show regression coefficients, as described in section A4. The sets of controls correspond with those in figure 3 of the main text.

Table A1: No consistent relationship between economic well-being and electrification

	(1)	(2)	(3)	(4)
dependent variable:	ln(income pc)	income pc	ln(consuption pc)	consumption pc
electrified	0.0621**	-2213.9***	0.0740***	-931.1*
	(2.59)	(-4.49)	(4.90)	(-2.44)
round 2 (2012) FE	0.347***	9704.0***	0.312***	7606.3***
	(27.56)	(19.57)	(34.71)	(24.33)
household FEs	✓	✓	✓	✓
n (households)	78,362	79,583	79,527	79,527

† two-sided $p < 0.10$, * $p < 0.05$, ** $p < 0.01$, *** $p < 0.001$. pc = "per capita." t-statistics in parentheses.

Table A2: Concentration-response function between change in coal plant exposure and cough

	(1)	(2)	(3)	(4)	(5)
sample:	ΔMW ≤ 630	ΔMW $\leq 1,000$	full	full	full
ΔGW coal plant capacity	0.0517†	0.0349†	0.0834*	0.0834*	
	(0.0312)	(0.0188)	(0.0363)	(0.0362)	
ΔGW coal plant capacity^2			-0.0587*	-0.0563*	
			(0.0255)	(0.0253)	
joint F test of quadratic			$p = 0.059$	$p = 0.064$	
$\ln(\Delta\text{MWcoal} + 1)$					0.00379†
					(0.00212)
2012 fixed effect	-0.0176***	-0.0173***	-0.0178***	-0.0205***	-0.0182***
	(0.00431)	(0.00428)	(0.00431)	(0.00569)	(0.00433)
village/PSU FEs	✓	✓	✓	✓	✓
full controls				✓	
n (households)	78,726	79,276	79,968	79,968	79,968

† two-sided $p < 0.10$, * $p < 0.05$, ** $p < 0.01$, *** $p < 0.001$. In each specification the dependent variable is the same respiratory health variable as in the main results. The full 79,968 household sample in columns 3 though 5 is the same as in the main results. 1 GW = 1,000 MW.

Table A3: Gaining a coal plant does not predict alternative measures of socio-economic status

	(1)	(2)	(3)	(4)	(5)	(6)
dependent var:	ln(income p.c.)	literate adult	no. of rooms	assets p.c.	electrified	piped water
gained coal plant	-0.0419	0.00712	0.0329	0.0188	-0.00323	-0.0118
	(0.0374)	(0.0105)	(0.0719)	(0.0651)	(0.0193)	(0.0208)
2012 FE	0.359***	0.0296***	0.0171	1.325***	0.108***	0.0372***
	(0.0131)	(0.00375)	(0.0236)	(0.0232)	(0.00684)	(0.00732)
household FE	✓	✓	✓	✓	✓	✓
constant	9.021***	0.759***	2.697***	1.184***	0.655***	0.229***
	(0.0191)	(0.00546)	(0.0344)	(0.0337)	(0.00995)	(0.0106)
n (households)	78,791	79,954	78,941	80,015	79,583	79,838

† two-sided $p < 0.10$, * $p < 0.05$, ** $p < 0.01$, *** $p < 0.001$. Each column reports a separate regression of the form $\text{SES}_{ipdt} = \beta \textit{gained coal}_{dt} + \alpha_{ipd} + \gamma_t + \varepsilon_{ipdt}$, where the dependent variable is as listed at the top of the column, α is a household fixed effect, and γ is a fixed effect for the 2012 survey round. The sample size varies very slightly due to item non-response across survey questions.

Table A4: Summary statistics about coal and non-coal plants gained, by type

	count of new plants			new capacity (MW)		
	mean	min	max	mean	min	max
coal	0.196	0	5	65.7	0	1,980
gas	0.047	0	9	7.5	0	1,530
hydro	0.128	0	16	8.5	0	1,520
naphtha	0.014	0	6	3.1	0	1,327
nuclear	0.017	0	2	7.1	0	1,080

Summary statistics reflect exposure of households in the IHDS, which are the units of observation. So, in the top left cell for example, the mean household lives is a district that gained 0.196 coal plants, which matches the summary statistic in Table 1 of the main text.

Table A5: Robustness check: Similar results from the IHDS individual recode

	(1)	(2)	(3)	(4)	(5)	(6)
dependent variable:	reported short term morbidity cough				diarrhea	fever
Δ coal plants	0.00516*	0.00445†	0.00430†		-0.00198	-0.000854
	(0.00250)	(0.00251)	(0.00251)		(0.00138)	(0.00259)
Δ coal capacity (GW)				0.0152*		
				(0.00681)		
ln(consumption p.c.)		-0.00788**	-0.0119**			
		(0.00118)	(0.00119)			
household size		0.00617**	0.00684**			
		(0.000260)	(0.000268)			
age × sex			✓			
2012 FE	-0.0350**			-0.0350**	0.00229†	0.0522**
	(0.00207)			(0.00207)	(0.00120)	(0.00231)
2012 × urban FEs		✓	✓			
PSU fixed effects	✓	✓	✓	✓	✓	✓
n (persons)	419,988	419,988	419,984	419,988	419,988	419,988

† two-sided $p < 0.10$, * $p < 0.05$, ** $p < 0.01$, *** $p < 0.001$. Data are taken from the "short term morbidity" section in the IHDS individual recode, so observations in this table only are individuals, not households. Coefficients are plausibly smaller in magnitude than in the main result because households contain multiple people; an individual-level coefficient of 0.005 would be comparable to a household-level coefficient of 0.025 if the average household has about 5 persons. Columns 5 and 6 repeat the functional form of column 1 while using diarrhea and fever as falsification tests, as in the main results.

Table A6: Robustness of balance table: Replicating Table 3 with all Table 4 specifications, Part 1

	(1)	(2)	(3)	(4)	(5)	(6)	(7)	(8)
model type:	OLS	OLS	OLS	OLS	OLS	OLS	OLS	logit
Panel A: Dependent variable is "daily milk right"								
additional coal plants	-0.000775	-0.00132	-0.00133			-0.00153	-0.00120	0.00845
	(0.0109)	(0.0108)	(0.0108)			(0.0108)	(0.0108)	(0.0357)
additional coal plants (top-coded)				0.000117				
				(0.0111)				
dichotomized additional coal plant					0.0159			
					(0.0200)			
additional non-coal plants						0.00278		
						(0.00609)		
additional coal plants × urban							0.00790	
							(0.0232)	
Panel B: Dependent variable is "colostrum right"								
additional coal plants	0.0254**	0.0262**	0.0261**			0.0256**	0.0260**	0.158***
	(0.00947)	(0.00952)	(0.00952)			(0.00951)	(0.00944)	(0.0319)
additional coal plants (top-coded)				0.0269**				
				(0.00981)				
dichotomized additional coal plant					0.0304			
					(0.0197)			
additional non-coal plants						0.00645		
						(0.00426)		
additional coal plants × urban							-0.00593	
							(0.0186)	
PSU (village/place) fixed effects	✓	✓	✓	✓	✓	✓	✓	✓
2012 fixed effect	✓							✓
urban × 2012 fixed effects		✓	✓	✓	✓	✓	✓	
full set of controls			✓	✓	✓	✓	✓	

† two-sided $p < 0.10$, * $p < 0.05$, ** $p < 0.01$, *** $p < 0.001$. For a complete description of each column, see the discussion in the text of Table 3; for a complete description of each dependent variable, see the discussion in the text of Table 4.

Table A7: Robustness of balance table: Replicating Table 3 with all Table 4 specifications, Part 2

	(1)	(2)	(3)	(4)	(5)	(6)	(7)	(8)
model type:	OLS	OLS	OLS	OLS	OLS	OLS	OLS	logit
Panel C: Dependent variable is "smoke bad for health right"								
additional coal plants	0.0486***	0.0491***	0.0491***			0.0488***	0.0483***	0.369***
	(0.0105)	(0.0104)	(0.0104)			(0.0104)	(0.0103)	(0.0351)
additional coal plants (top-coded)				0.0504***				
				(0.0108)				
dichotomized additional coal plant					0.123***			
					(0.0213)			
additional non-coal plants						0.00407		
						(0.00325)		
additional coal plants × urban							-0.0370+	
							(0.0208)	
Panel D: Dependent variable is "diarrhea requires fluids right"								
additional coal plants	0.00852	0.00982	0.00961			0.00760	0.00910	0.0421
	(0.0124)	(0.0125)	(0.0125)			(0.0125)	(0.0128)	(0.0279)
additional coal plants (top-coded)				0.0113				
				(0.0129)				
dichotomized additional coal plant					0.0285			
					(0.0265)			
additional non-coal plants						0.0229**		
						(0.00851)		
additional coal plants × urban							-0.0299	
							(0.0297)	
PSU (village/place) fixed effects	✓	✓	✓	✓	✓	✓	✓	✓
2012 fixed effect	✓							✓
urban × 2012 fixed effects		✓	✓	✓	✓	✓	✓	
full set of controls			✓	✓	✓	✓	✓	

† two-sided $p < 0.10$, * $p < 0.05$, ** $p < 0.01$, *** $p < 0.001$. For a complete description of each column, see the discussion in the text of Table 3; for a complete description of each dependent variable, see the discussion in the text of Table 4.

Table A8: Robustness of balance table: Replicating Table 3 with all Table 4 specifications, Part 3

	(1) OLS	(2) OLS	(3) OLS	(4) OLS	(5) OLS	(6) OLS	(7) OLS	(8) logit
Panel E: Dependent variable is "knows health provider (doctor or nurse)"								
additional coal plants	0.00310	0.00865	0.00811			0.00821	0.00856	0.00162
	(0.0115)	(0.0114)	(0.0114)			(0.0114)	(0.0112)	(0.0255)
additional coal plants (top-coded)				0.00846				
				(0.0119)				
dichotomized additional coal plant					0.0468+			
					(0.0251)			
additional non-coal plants						-0.00111		
						(0.00413)		
additional coal plants × urban							0.0143	
							(0.0222)	
Panel F: Dependent variable is "LPG or other clean fuel stove"								
additional coal plants	-0.00597	-0.000722	-0.000904			-0.000550	-0.000762	0.00344
	(0.00575)	(0.00572)	(0.00579)			(0.00580)	(0.00584)	(0.0389)
additional coal plants (top-coded)				-0.000889				
				(0.00602)				
dichotomized additional coal plant					-0.00518			
					(0.0155)			
additional non-coal plants						-0.00422		
						(0.00308)		
additional coal plants × urban							0.00461	
							(0.0138)	
PSU (village/place) fixed effects	✓	✓	✓	✓	✓	✓	✓	✓
2012 fixed effect	✓							✓
urban × 2012 fixed effects		✓	✓	✓	✓	✓	✓	
full set of controls			✓	✓	✓	✓	✓	

† two-sided $p < 0.10$, * $p < 0.05$, ** $p < 0.01$, *** $p < 0.001$. For a complete description of each column, see the discussion in the text of Table 3; for a complete description of each dependent variable, see the discussion in the text of Table 4.
